# Effectiveness and safety of Tai Chi for anxiety disorder of COVID-19: A protocol of systematic review and meta-analysis

**DOI:** 10.1097/MD.0000000000030992

**Published:** 2022-10-14

**Authors:** Shiqiang Zhang, Luwen Zhu, Runyu Liang, Xia Yin, Ruoyu Wang, Xiyuan Ma, Hongyu Li, Qiang Tang

**Affiliations:** a Heilongjiang University of Chinese Medicine, Harbin, China; b The Second Affiliated Hospital of Heilongjiang University of Chinese Medicine, Harbin, China.

**Keywords:** anxiety disorders, COVID-19, meta-analysis, protocol, Tai Chi

## Abstract

Anxiety disorders pose a significant threat to the clinical rehabilitation of patients with coronavirus disease 2019 (COVID-19). Tai Chi is a therapeutic exercise that can be used to treat anxiety disorders. We aim to conduct a systematic review and meta-analysis to evaluate the effectiveness and safety of Tai Chi for treating patients with anxiety disorders caused by COVID-19.

**Methods:**

The PubMed, EMBASE, Cochrane Library, China National Knowledge Infrastructure, Chinese Biomedical Literature, Wan Fang, and Chinese Clinical Trial Registry databases will be searched for reports of randomized controlled trials on Tai Chi for the treatment of anxiety disorders caused by COVID-19, published from December 1, 2019, to August 22, 2022. Two researchers will screen the articles and extract the relevant information.

**Results:**

The results will provide a systematic overview of the current evidence on the use of Tai Chi to treat anxiety disorders caused by COVID-19 among patients.

**Conclusion:**

The conclusions of this study will help clarify whether Tai Chi is effective and safe for treating anxiety disorders caused by COVID-19.

## 1. Introduction

The COVID-19 pandemic is a major global public health challenge. Since the outbreak of COVID-19 in Wuhan, China, in December 2019, the number of confirmed cases and deaths due to COVID-19 worldwide has exceeded 525 million and 6.2 million (as of August 2022), respectively, and continues to increase.^[1]^ The severe acute respiratory syndrome coronavirus 2 (SARS-CoV-2), which causes COVID-19, is a novel virus; thus, the general population is highly susceptible and has no specific immunity against it. In addition, SARS-CoV-2 is mutating frequently and rapidly, making the development of established treatment regimens for COVID-19 challenging. Alpha, beta, gamma, delta,^[2]^ and omicron variants of SARS-CoV-2 have been identified so far.^[3]^ Therefore, although the existing therapeutic regimens for COVID-19 generally alleviate the symptoms of the disease, the rapid and frequent development of new variants of SARS-CoV-2^[4,5]^ results in the recurrence of COVID-19.

The survey of patients with COVID-19 showed that more than 40% of the respondents experienced some degree of anxiety and that 18.6% of those who experienced anxiety also had moderate to severe depression or stress.^[6]^ Patients with COVID-19 generally show signs of emotional distress, including anxiety,^[7]^ memory and attention loss, sleep disorders, communication disorders, post-traumatic stress disorder, and other mental health problems.^[8]^ Regarding social activities, patients with COVID-19 often experience anxiety due to the adverse effects of the COVID-19 pandemic, such as reduced social contact, loneliness, incomplete recovery of physical health, or unemployment caused by the disease.^[9]^

Post-COVID-19 anxiety disorder is a health condition that usually requires long-term treatment, which has a long-term negative impact on the physiological and psychological health of patients.^[10]^ With the steady increase in the number of patients with COVID-19, the negative impact of post-COVID-19 anxiety disorders on individuals, families, and the society is emerging,^[11]^ and has become a health problem that cannot be ignored. Thus, post-COVID-19 anxiety disorder has become a hot topic in the COVID-19 discourse globally.

It is important to understand the symptoms and effects of anxiety disorders in patients after COVID-19 to reduce and avoid negative effects and improve symptom management in such patients. Research has shown that Tai Chi can be used to treat anxiety, depression, traumatic stress disorders, and other mental health conditions.^[12,13]^ Tai Chi is characterized by soft movements, concentrated thoughts, command of movements with thoughts, coordinated breathing, and control of hand-eye movements with breathing. It requires coordination of the hands, feet, head, and eyes, and internal and external cultivation.^[14]^ Tai Chi cultivates the mind; seeks harmony between man and nature; and has a good effect on the adjustment of the states of the respiratory system, nervous system, and internal organs.^[15,16]^ It focuses on the internal essence and spirit, thus playing a role in cultivating the mind, relieving anxiety and depression,^[17]^ and promoting a healthy state of mind. Therefore, we will perform a systematic review and meta-analysis to explore the effectiveness and safety of Tai Chi in treating anxiety disorders caused by COVID-19 to provide a reference for treating health crises caused by post-COVID-19 anxiety disorders.

## 2. Methods

### 2.1. Protocol registration

This research program was registered in PROSPERO (registration number: CRD42022320766). The protocol report was based on the Preferred Reporting Items for Systematic Review and Meta-Analysis Protocols 2015 statement.^[18]^

### 2.2. Inclusion criteria

#### 2.2.1. Participants.

COVID-19 patients with anxiety disorders will be included. There will be no restriction on the age, race, and country of the participants.

#### 2.2.2. Interventions.

The experimental group will include patients who underwent Tai Chi, including modified Tai Chi. The control group will include patients who received Western or Chinese herbal medicine, except Tai Chi.

#### 2.2.3. Outcomes.

The primary outcome will be changes in Hamilton Anxiety Scale^[19]^ and Self-Rating Anxiety Scale^[20]^ scores after treatment. The secondary outcome indicators will be Liebowitz Social Anxiety Scale^[21]^ and Short Form 36 questionnaire scores. SF-36 is used to measure an individual’s quality of life.^[22]^

Data on adverse events and the safety of the included studies will be utilized for safety analysis.

#### 2.2.4. Studies.

We will include randomized controlled trials (RCTs).

### 2.3. Search strategy

We will search the electronic databases of PubMed, EMBASE, Cochrane Library, China National Knowledge Infrastructure, Chinese Biomedical Literature Database, Wan Fang, and the Chinese Clinical Trial Registry for RCTs on Tai Chi for the treatment of anxiety disorders caused by COVID-19. Relevant articles published from December 1, 2019, to August 22, 2022, will be included. The search strategy for PubMed is presented in Table 1.

**Table 1:** PubMed search strategy.

Search	Query
#1	“Tai Ji”[Mesh]
#2	“Tai-ji”[Title/Abstract] OR “Tai Chi”[Title/Abstract] OR “Tai Ji Quan”[Title/Abstract] OR “Quan, Tai Ji”[Title/Abstract] OR “Taiji”[Title/Abstract] OR “Taijiquan”[Title/Abstract] OR (Tai Chi Chuan[Title/Abstract]
#3	#1 OR #2
#4	“COVID-19”[Mesh]
#5	“COVID 19”[Title/Abstract] OR “Coronavirus Disease 2019”[Title/Abstract] OR “SARS-CoV-2 Infection”[Title/Abstract] OR “SARS CoV 2 Infection”[Title/Abstract] OR “2019 Novel Coronavirus Disease”[Title/Abstract] OR “2019 Novel Coronavirus Infection”[Title/Abstract] OR “2019-nCoV Disease”[Title/Abstract] OR “COVID-19 Virus Infection”[Title/Abstract] OR “Severe Acute Respiratory Syndrome Coronavirus 2 Infection”[Title/Abstract] OR “SARS Coronavirus 2 Infection”[Title/Abstract] OR “COVID-19 Virus Disease”[Title/Abstract] OR “2019-nCoV Infection”[Title/Abstract]
#6	#4 OR #5
#7	“Anxiety Disorders”[Mesh]
#8	“Anxiety Disorder”[Title/Abstract] OR “Anxiety Neuroses”[Title/Abstract] OR “Anxiety States, Neurotic”[Title/Abstract] OR “Anxiety State, Neurotic”[Title/Abstract] OR “Neurotic Anxiety State”[Title/Abstract] OR “Neurotic Anxiety States”[Title/Abstract]
#9	#7 OR #8
#10	“Randomized Controlled Trial”[Publication Type] OR “randomized”[Title/Abstract] OR “placebo”[Title/Abstract]
#11	#3 AND #6 AND # 9 AND #10

COVID-19 = coronavirus disease 2019.

### 2.4. Selections of studies

**Figure 1.**
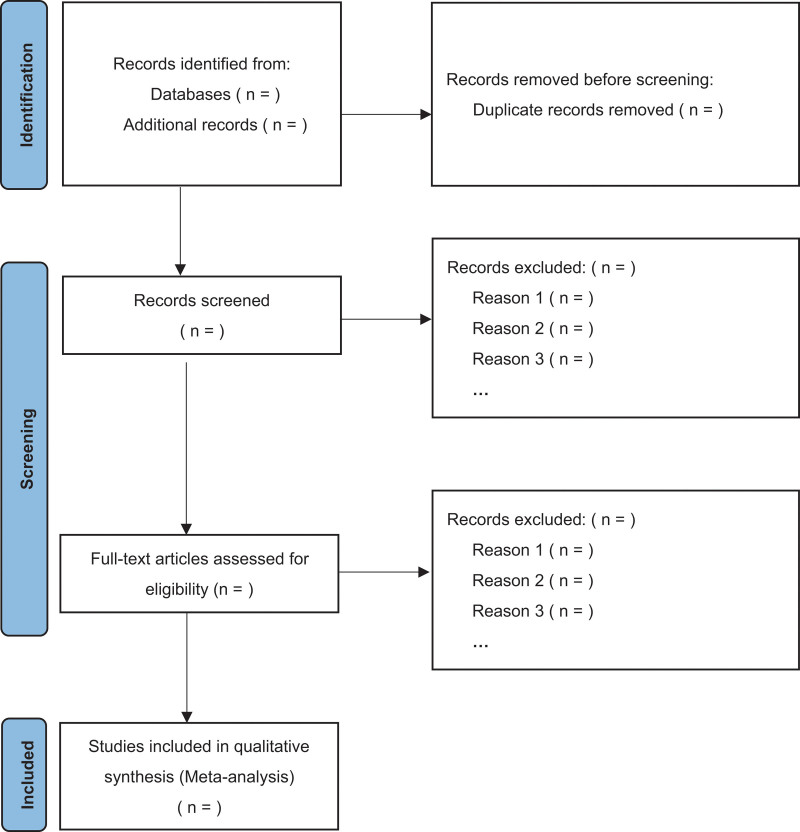
The study selection process.

Two reviewers (S-QZ and XY) will independently review and screen the studies according to the inclusion and exclusion criteria of the review. First, the reviewers will utilize EndNote X9 software to exclude duplicate articles. Thereafter, they will exclude obviously irrelevant literature by reading the titles and abstracts of the articles. They will then screen the remaining articles by reading their full texts and provide the reasons for excluding ineligible studies. The selection process is shown in Figure 1.

### 2.5. Data collection and management

Two reviewers (H-YL and X-YM) will independently screen the literature and extract the data from the included studies. The collected data will include the information of each study (year of publication, first author, sample size, age and sex of participants, course of the disease, intervention, control, course of treatment, primary outcome, and secondary outcome) and its results (results of outcomes, adverse events, and safety). If there is any disagreement between the two reviewers during the screening, they will consult a third reviewer (QT) for the final decision.

### 2.6. Assessment of risk of bias

Two researchers (R-YL and R-YW) will separately evaluate the methodological qualities of the literature using the Cochrane Collaboration’s Risk of Bias tool, which includes 7 items. The risk of bias in each aspect will be assessed, and the results will be categorized into three grades: low risk, unclear risk, and high risk.

### 2.7. Data syntheses

#### 2.7.1. Data synthesis and assessment of heterogeneity.

RevMan software 5.4 will be used for statistical analyses. We will analyze continuous variables using mean differences or standard mean differences with 95% confidence intervals. *I*^2^ is used to evaluate the heterogeneity of literature statistics. When *I*^2^ < 50% indicates low heterogeneity, the fixed effect model will be used for analysis. When *I*^2^ ≥ 50%, indicating high heterogeneity, it is necessary to determine the cause of heterogeneity and select appropriate methods to reduce heterogeneity.

#### 2.7.2. Assessment of reporting bias.

We will utilize a funnel plot to assess reporting bias if the included studies are more than ten. An asymmetrical funnel plot indicates the existence of publishing bias.

#### 2.7.3. Subgroup analysis.

If significant heterogeneity exists, subgroup analysis will be performed according to different courses of treatment, levels of anxiety, or types of Tai Chi.

#### 2.7.4. Sensitivity analysis.

We will conduct a sensitivity analysis to assess the robustness and reliability of the results by excluding low-quality studies and focusing on missing data.

#### 2.7.5. Grading the quality of evidence.

We will use the Grading of Recommendations Assessment, Development, and Evaluation Reliability Study system to assess the quality of the obtained evidence.

#### 2.7.6. Dealing with missing data.

We will try to contact the corresponding author of any study with missing data to obtain the missing information. If the corresponding author cannot be reached, we will use the available data for synthesis, and the potential impact of the missing information will be reviewed.

#### 2.7.7. Ethics and dissemination.

No ethical approval is needed for this type of systematic review as the data is reviewed retrospectively. This systematic review and meta-analysis will be published in a peer-reviewed journal.

## 3. Discussion

The COVID-19 pandemic has had a significant impact on the mental health of patients and healthcare workers. Patients diagnosed with COVID-19 require isolation, and during isolation, the patients tend to experience fear, loneliness, sadness, and other negative emotions, which result in anxiety.^[23]^ Although several patients with COVID-19 recover, they experience serious psychological effects of the disease, including anxiety.^[24]^ Tai Chi is a traditional Chinese rehabilitation therapy with a long history. It focuses on self-cultivation, mood improvement, and strengthening of the body.^[25]^ It has been found that Tai Chi training can not only strengthen the body and improve the body’s immunity and lung function but also improve anxiety and depression.^[26]^ Our systematic review and meta-analysis can serve as a reference for the clinical treatment and improvement of the psychological status of patients with COVID-19.

## Author contributions

**Assessment of risk bias:** Runyu Liang, Ruoyu Wang.

**Conceptualization:** Shiqiang Zhang, Luwen Zhu.

**Data curation:** Hongyu Li, Xiyuan Ma.

**Formal analysis:** Shiqiang Zhang, Hongyu Li.

**Funding:** Luwen Zhu.

**Investigation:** Shiqiang Zhang, Xia Yin.

**Project administration:** Qiang Tang.

**Resources:** Shiqiang Zhang.

**Software:** Shiqiang Zhang.

**Writing – original draft:** Hongyu Li, Shiqiang Zhang.

**Writing – review & editing:** Hongyu Li, Qiang Tang.
